# Forecasting of stage-discharge in a non-perennial river using machine learning with gamma test

**DOI:** 10.1016/j.heliyon.2023.e16290

**Published:** 2023-05-13

**Authors:** Dinesh Kumar Vishwakarma, Alban Kuriqi, Salwan Ali Abed, Gottam Kishore, Nadhir Al-Ansari, Kusum Pandey, Pravendra Kumar, N.L. Kushwaha, Arif Jewel

**Affiliations:** a Department of Irrigation and Drainage Engineering, G.B. Pant University of Agriculture and Technology, Pantnagar, Uttarakhand, 263145, India; b CERIS, Instituto Superior T′ecnico, University of Lisbon, 1649–004, Lisbon, Portugal; c Civil Engineering Department, University for Business and Technology, Pristina, Kosovo; d College of Science, University of Al-Qadisiyah, Qadisiyyah, 58002, Iraq; e ICAR-Central Institute of Agricultural Engineering, Bhopal, Madhya Pradesh, India; f Civil, Environmental and Natural Resources Engineering, Lulea University of Technology, 97187, Lulea, Sweden; g Department of Soil and Water Conservation Engineering, Punjab Agriculture University, Ludhiana, Punjab 141004, India; h Department of Soil and Water Conservation Engineering, G.B. Pant University of Agriculture and Technology, Pantnagar, Uttarakhand, 263145, India; i Division of Agricultural Engineering, ICAR-Indian Agricultural Research Institute, New Delhi, 110012, India; j Centre for Irrigation and Water Management, Rural Development Academy (RDA), Bogura, 5842, Bangladesh; k G. B. Pant National Institute of Himalayan Environment, Garhwal Regional Center, Srinagar, Uttarakhand 246174, India

**Keywords:** Rating curve, GRG technique, Stage-discharge forecasting, Machine learning, Logistic regression

## Abstract

Knowledge of the stage-discharge rating curve is useful in designing and planning flood warnings; thus, developing a reliable stage-discharge rating curve is a fundamental and crucial component of water resource system engineering. Since the continuous measurement is often impossible, the stage-discharge relationship is generally used in natural streams to estimate discharge. This paper aims to optimize the rating curve using a generalized reduced gradient (GRG) solver and the test the accuracy and applicability of the hybridized linear regression (LR) with other machine learning techniques, namely, linear regression-random subspace (LR-RSS), linear regression-reduced error pruning tree (LR-REPTree), linear regression-support vector machine (LR-SVM) and linear regression-M5 pruned (LR-M5P) models. An application of these hybrid models was performed and test to modeling the Gaula Barrage stage-discharge problem. For this, 12-year historical stage-discharge data were collected and analyzed. The 12-year historical daily flow data (m^3^/s) and stage (m) from during the monsoon season, *i.e.,* June to October only from 03/06/2007 to 31/10/2018, were used for discharge simulation. The best suitable combination of input variables for LR, LR-RSS, LR-REPTree, LR-SVM, and LR-M5P models was identified and decided using the gamma test. GRG-based rating curve equations were found to be as effective and more accurate as conventional rating curve equations. The outcomes from GRG, LR, LR-RSS, LR-REPTree, LR-SVM, and LR-M5P models were compared to observed values of daily discharge based on Nash Sutcliffe model efficiency coefficient (NSE), Willmott Index of Agreement (d), Kling-Gupta efficiency (KGE), mean absolute error (MAE), mean bias error (MBE), relative bias in percent (RE), root mean square error (RMSE) Pearson correlation coefficient (PCC) and coefficient of determination (R^2^). The LR-REPTree model (combination 1: NSE = 0.993, d = 0.998, KGE = 0.987, PCC(r) = 0.997, and R^2^ = 0.994 and minimum value of RMSE = 0.109, MAE = 0.041, MBE = −0.010 and RE = −0.1%; combination 2; NSE = 0.941, d = 0.984, KGE = 0. 923, PCC(r) = 0. 973, and R^2^ = 0. 947 and minimum value of RMSE = 0. 331, MAE = 0.143, MBE = −0.089 and RE = −0.9%) performed superior to the GRG, LR, LR-RSS, LR-SVM, and LR-M5P models in all input combinations during the testing period. It was also noticed that the performance of the alone LR and its hybrid models (i.e., LR-RSS, LR-REPTree, LR-SVM, and LR-M5P) was better than the conventional stage-discharge rating curve, including the GRG method.

## 1 Introduction

Stream discharge is important and basic data required in hydraulic and hydrologic studies. It provides information that helps control and manage flood frequency analysis, sediment studies, water demand, water available resources sustainably planning, and computation of standard project flood [[1], [2], [3]]. It is essential to measure stream flow accurately to determine peak discharge to hydraulic design structures that are both safe and economically feasible [4,5]. The development of rating curves is still an area of interest for hydrologists, and it has been studied interchangeably by them [3]. Stage-discharge analysis of a river can be carried out by consistently assessing discharge and stage using a current meter and other techniques (*i.e.,* velocity measurements and dilution methods). The results could be statistical analyses to develop rating curves [6]. The relationship stage-discharge, specific to a station along the stream, can be calculated using mathematical relationships [7]. Once the stage-discharge relationship is set up, readings need only be taken off stage because the discharge may be recorded via a stage-discharge curve [2,7,8]. It is necessary to analyze the streams-discharge relationship for flood routing and damage control, sediment analysis, providing habitats for biological communities, and sustaining high water quality [[9], [10], [11]]. These properties of stream-discharge relation benefit the people living within the watershed or basin [12]. The uncontrolled discharge may convert into disasters and severely impact society regarding socio-economic casualties if not handled carefully [13,14]. Therefore, stage-discharge management is crucial for preventing and/or mitigating adverse impacts and should include structural and nonstructural measures [15,16].

A linear MLR model is employed to predict the most common output-input variables, and these multiple variables are linearly related [17]. Different analytical models were used to forecast stage-discharge for monitoring the water resource activities at the basin level, like flood routing, flood mitigation and protection, drought assessment, and optimization of reservoir activities for agriculture, electricity generation, and drinking water supply [[18], [19], [20], [21]]. Additionally, the polynomial equations that characterize the stage-discharge relationship fail to effectively forecast the peak values [[23], [24], [25]]. Usually, the stage-discharge observations are made manually during the day, and flood peaks often strike at night, adding to the discharge uncertainty [22,23]. There is numerous formula available for stage-discharge calculation. However, they have hysteresis issues, particularly when a high-flow forecast is required [4,24]. Discharge measurement is important for designing hydraulic structures and their safe downstream passage. Different empirical equations are available for lower discharge, i.e., Manning's. Therefore, simulations are run in a controlled laboratory environment to investigate the impact of their various geometry and hydraulic parameters on flow characteristics [25].

The artificial intelligence and machine learning models, sometimes known as “black-box” or “data-driven” models, are based on time series data [26,27]. These models can capture the complex non-linear relations between input and output variables during forecasting [[28], [29], [30]]. Also, these models are flexible enough to predict hydrological problems with high efficiency [[31], [32], [33]]. Machine learning and artificial intelligence models have become very popular in recent decade [[34], [35], [36], [37], [38]]. Forecasting of the stream discharge various models such as multiple-linear regression (MLR) [2,[39], [40], [41], [42]], rating curve [[43], [44], [45], [46], [47]], wavelet-based MLR (WMLR) [48,49], support vector machine (SVM) [39,44,[50], [51], [52], [53]], artificial neural network (ANN) [45,[53], [54], [55], [56], [57]], wavelet-based artificial neural network (WANN) [2,39,58], adaptive neuro-fuzzy inference system (ANFIS) [[59], [60], [61]], wavelet-based support vector machine (WSVM) [39,62], wavelet–bootstrap–ANN (WBANN) [48,63], M5-model trees [46,64], random forest (RF) [65], ARIMA [65,66], gene expression programming (GEP) [32,67,68], genetic algorithm (GA) [3,33,69], genetic programming (GP) [32], Bagged M5P [65], integrating long-short-term memory (LSTM) [69,70], wavelet–bootstrap–multiple linear regression (WBMLR) [48], Fuzzy logic and fuzzy neuro systems [59,71] multi-objective evolutionary neural network (MOENN) [59], and Gaussian process regressions (GPR) [47], among others have emerged as viable tools for discharge estimation. Aggarwal et al. [53] applied SVM and ANN algorithms to predict the stage-discharge in the Mahanadi River, India. It is shown that it is challenging to outperform the persistence model over a shorter forecasting horizon.

In addition, the results revealed that the SVM model was able to forecast stage-discharge over a longer time period with more accuracy than the other models [53,58]. A comparison between the ANFIS and the ARIMA-based modeling of the day-ahead streamflow of the Klang River, Malaysia, was carried out by Galavi et al. [66]. They found that the ANFIS outperformed the ARIMA model for day-ahead streamflow forecasting, showing that the ANFIS was more efficient than the ARIMA model. However, Hipni et al. [70] found that for prediction the level of water in a dam on a daily basis for the Klang reservoir, Malaysia, SVM, provides better prediction than the ANFIS model. Pham et al. [72] proposed a hybrid model based on a ML algorithm, MLP with intelligent water drop optimization algorithm (MLP-IWD) for the predictions of the monthly stream flow for the Vu Gia Thu Bon river basin, South Central Vietnam, and considers only the lagged flow rate (i.e., 36 months) as input.

Norouzi et al. [73] observed that the multi-layer perceptron (MLP) generates reliable findings as compared to radial basis function networks (RBF) and SVM with different kernel functions. The study concluded that MLP predicted precisely labyrinth weirs' discharge coefficient (Cd) with quarter-round crests. Another study was carried out by Kumar et al. [58] to predict the daily stage-discharge correlation in Burhabalang River Basin, Orissa, India, and select a wavelet-based ANN (WANN) model and SVM optimization technique with a linear and radial basis kernel function. According to the study, the number of input variables has a significant impact on the computation process, therefore making it extremely time-consuming, difficult to comprehend, and giving an inadequate result when the number of input variables is increased. Therefore, this study aims to carry out the researchers as water resources planners to set the new analytical models for potential applications in solving flood forecasting and mitigation problems in the area of hydrology as well as hydraulics. Birbal et al. [67] predicted the stage-discharge relationship with GEP model. The GEP model constructed the discharge rating curve (SRC) exceptionally well.

A number of wavelet, empirical model, and ensemble empirical mode decomposition (EEMD)-based GPR models were also tested and compared by Roushangar et al. [47] to modeling the stages-discharges at consecutive hydrometric. The results of the study indicate that the integrated WT and EEMD-GPR models have higher accuracy than conventional approach. The study reported that data processing enhanced the model capability by 30 and 45%. Nevertheless, it was noticed that machine learning-based algorithms usually generate reliable findings; some remain under-utilized for estimating stage-discharge relationships. As a result of the recent applications of efficient machine learning models to simulate several hydrologic and hydraulic challenges, we were compelled to investigate the applicability of related methods to model this relationship.

In light of the above-mentioned state-of-the-arts, the broader scientific literature, as well as the author's understanding, there have been no studies that have explored the hybridization of linear regression (LR) with other machine learning techniques *i.e.,* linear regression-random subspace (LR-RSS), linear regression-reduced error pruning tree (LR-REPTree), linear regression-support vector machine (LR-SVM) as well as linear regression-M5 pruned models for forecasting stage-discharge relationships,. Many researchers have applied machine learning algorithms and compared the performances [[74], [75], [76], [77], [78], [79], [80]] but have not explored the hybrid algorithms for the study stations. Therefore, this study aims to develop the hybrid models of LR with other machine learning algorithms so that the performance of the LR algorithm may be enhanced for forecasting the rating curve and discharge prediction using hydrological data. This study also compared the performance of developed hybrid models with conventional stage-discharge rating curves and Generalized Reduced Gradient (GRG).

## 2 Methodology

### 2.1 Study area and data collection

**Fig. 1:**
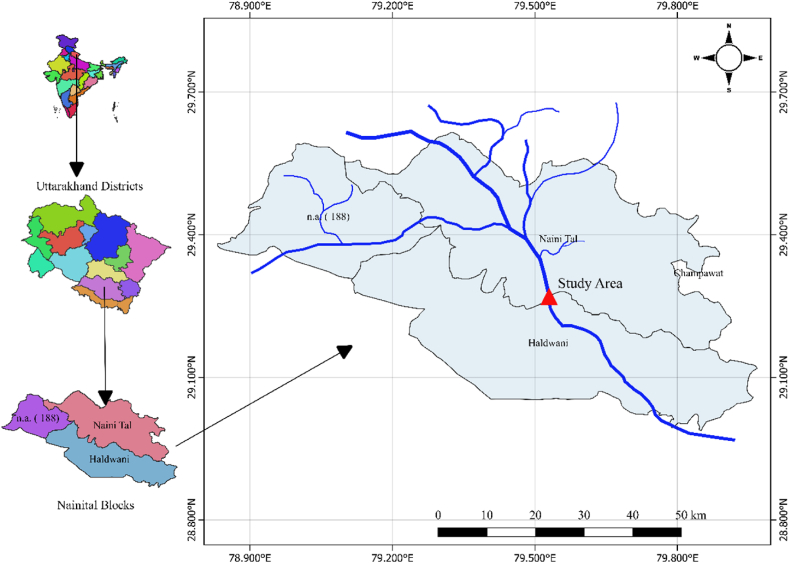
The location map of the Gaula Barrage is located at Kathgodam near Haldwani (Nainital), Uttarakhand.

An investigation was conducted on the Gaula Barrage (also known as Gola Barrage) in the steep town of Kathgodam, which is near Haldwani (Nainital district, Uttarakhand) in the hills of the Himalayas. The Gaula Barrage is a vital water source used to irrigate the Bhabhar fields. It is located at latitudes 29°16′18″ N and longitudes 79°32′51″E. The area is surrounded by subtropical to sub-humid climates and is located at a height of 554 m above sea level. Fig. 1 shows the location of the Gaula River basin. In the area, the mean annual precipitation is 2095 mm, and the maximum amount of precipitation each year occurs between June and October, when the rainiest weather occurs.

**Fig. 2:**
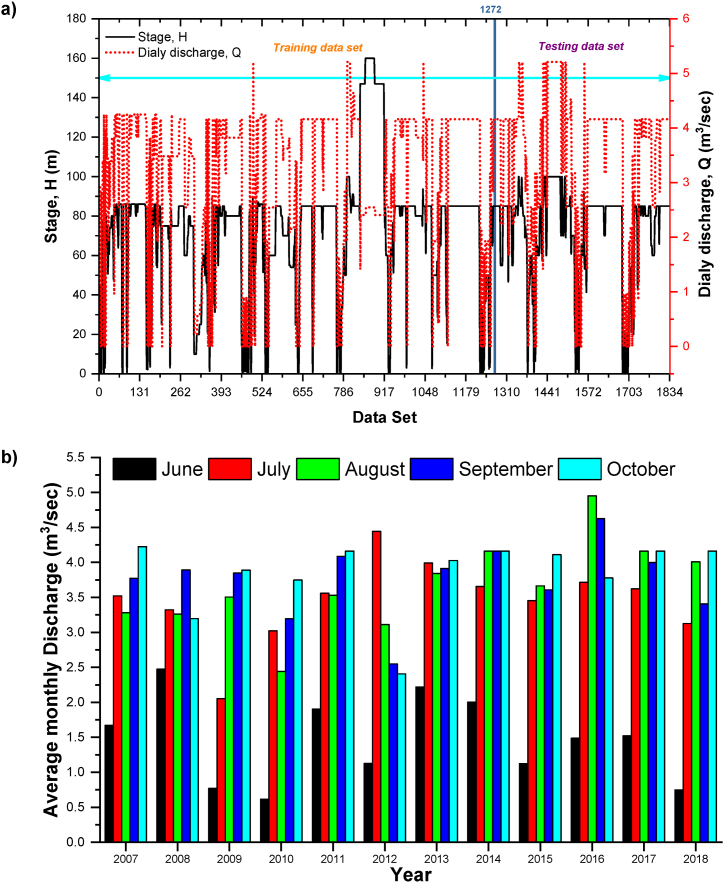
a) Daily and b) Monthly discharge at Gaula barrage.

**Fig. 3:**
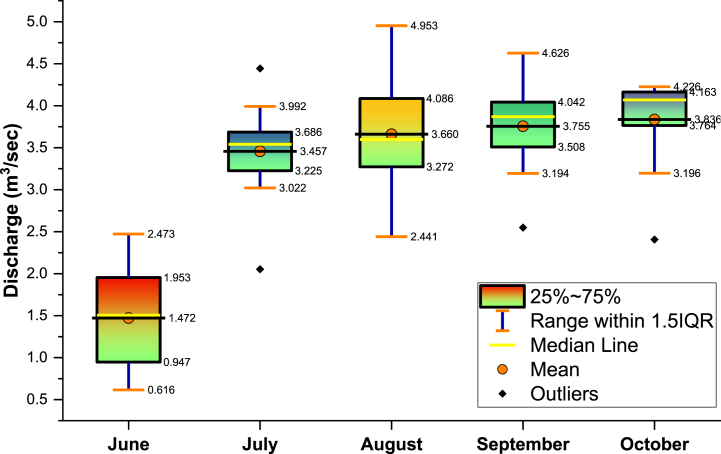
Visualizing the box and whisker plot of average monthly discharge at Gaula barrage.

**Fig. 4:**
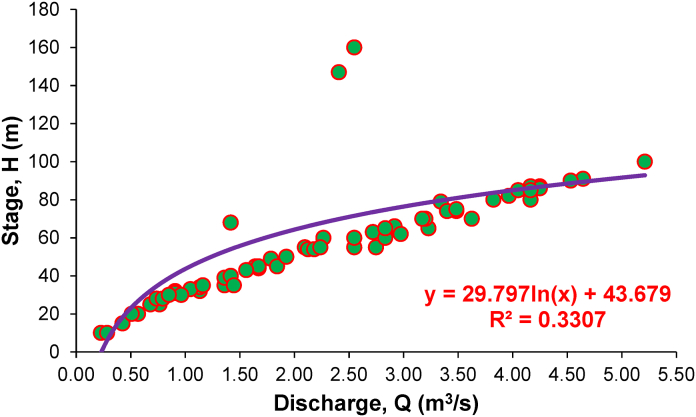
Rating curve of the stage-discharge relationship at the study site.

In the present study, stage-discharge models were used for the Gaula Barrage site, located on the Gaula River, during the monsoon season, which is between June and October, and 12 years of data were used for both stage (*H*) and discharge (*Q*) during this period. The whole data were separated into two categories: training data for calibrating the model and testing data for validating the model.70% of the data (1284 days) were used as training data for the model and further 30% of the data (550 days) was used for testing purposes. i.e., the training period (03/06/2007 to 01/08/2015) and testing period (02/08/2015 to 31/10/2018) (Fig. 2(a-b)). The characteristics statistics and range of discharge (*Q*) and head (*H*) at gauging stations are shown in Table 1, and visualizing the box and whisker plot of average monthly discharge is in Fig. 3. The significant skewness coefficient has shown that the model's efficacy has been significantly adversely affected. The minimal skewness coefficients for the specified station's calibration and validation coefficients indicate this location has found low calibrated and validated skewness coefficients. These statistical characteristics represent variability as the variation of data varies with time. Using the same statistical population in training and testing subsets was necessary, and the data had to be cross-validated. A high skewness coefficient considerably negatively influences the model's ability to predict future outcomes. A rating curve of the stage-discharge relationship at the study site is shown in Fig. 4 along with the discharge curve.

**Table 1:** Characteristics statistics and range of discharge (*Q*) and head (*H*) at gauging stations.

Statistical Parameter	Entire data		Training data		Testing data	
	Q (m^3^/sec)	H (m)	Q (m^3^/sec)	H (m)	Q (m^3^/sec)	H (m)
Mean	3.24	73.40	3.12	73.45	3.52	73.29
Standard Error	0.03	0.93	0.04	1.26	0.06	1.05
Median	3.82	85.00	3.82	80.00	4.16	85.00
Mode	4.16	85.00	4.16	85.00	4.16	85.00
Standard Deviation	1.39	39.89	1.39	44.94	1.36	25.03
Kurtosis	0.34	141.89	0.13	126.74	0.93	2.52
Skewness	−1.15	7.17	−1.15	7.37	−1.25	−1.74
Minimum	0.00	0.00	0.00	0.00	0.00	0.00
Maximum	5.21	85.00	5.21	85.00	5.21	85.00

### 2.2 Stage-discharge rating curve

During floods, continuous discharge measurement is time-consuming, costly, and impractical; therefore, most streams establish a relationship between stage and discharge by indirect means [81,82]. With the help of gauge data and empirical knowledge, hydrologists can define stage-discharge relationships for channel sections and reach using RCs, representing hydraulic behavior [83]. Graphs illustrating this relationship are known as rating curves (Fig. 4).

An accurate description of the actual behavior of the studied reach of the river requires knowledge of the wide range of historical hydrological conditions data between extreme discharges and stages. Several factors influence discharge through a river section, including channel characteristics, flow features, the slope of the bed, and many others.

Nevertheless, quantification of all these factors is not feasible [84,85]. The discharge of a gauging site can easily be determined from the observed stages once the rating curve has been established. This relationship has been shown previously in hydrological studies to be as follows:

(1) $$Q=KH^{b}$$

*Q* stands for the discharge at the river reach cross-section, *H* stands for the gauge height, and *K* and *b* are constant parameters in the equation. Later eq. (1) has been modified [86], and the term *H* can be written as *H = (H-a)*, where H is the gauge height of the water surface and stands for the river bed elevation;

(2) $$Q=K{\left(H-a\right)}^{b}$$

In Eq. (2), the constant *a* corresponds to the gauge height for zero discharge in the stream. In general, graphical methods estimate a value since it is a hypothetical parameter that cannot be directly determined in the field [87]. For estimating the value, *a* graphical method is used. Traditionally, regression is used to determine the best-fit value for K and n. Eq. (2) can be solved and may be written in logarithmic as:

(3) logQ=blog(H−a)+logK

Eq. (3) may be expressed as:

(4) Y=AX+B

where *Y* = log *Q*; A = *n*; *X* = log (*G* − *a*); *B* = log *K*.

The values of A and B in Eq. (4) can be calculated by regression analysis. Using regression analysis, finding the value of K and b parameters is easy. However, it is difficult and tedious to find the value of *a*. It is possible to estimate rating curve parameters directly using optimization techniques instead of going through such a tedious process. The present study estimated the rating curve parameters using the Generalized Gradient (GRG) technique.

#### 2.2.1 Generalized reduced gradient technique (GRG)

In 1978, Lasdon et al. [88] developed a non-linear optimization code called GRG solver. In Microsoft Excel, a GRG solution can be used to determine the optimum values of parameters for both linear and non-linear equations. MS Excel's solver methods include LP solver (linear programming solver) that solves and optimizes linear equations, GRG solver (Generalized Reduced Gradient), and evolutionary solvers that solve non-linear equations. This study uses the GRG method to estimate the parameters of the rating curve. Eq. (2) was used to calculate discharge for each stage based on the assumed values of the variables. An objective function was optimized using GRG and appropriate bounds on rating curve parameters to determine optimal values for rating curve parameters. The objective function was to minimize the sum of squares of differences between observed and predicted discharges as shown in Eq. (5):

(5) $$\mathrm{Min}\;SSQ=\sum_{i=1}^{N}{\left(Q_{Obs}^{i}-Q=K{\left(H-a\right)}^{b}\right)}^{2}$$

Over the past few years, Microsoft Excel has been used extensively in various engineering fields. Additionally, the GRG solver has been used to calculate infiltration equation parameters [84,89,90], parameter estimation of the non-linear Muskingum routing models [91], optimal unit hydrograph of watersheds [92], and rating curve, among others [3,93]. A GRG solver was also used to estimate intensity duration frequency (IDF) parameters by Zakwan [94].

## 3 Machine learning models used

**Fig. 5:**
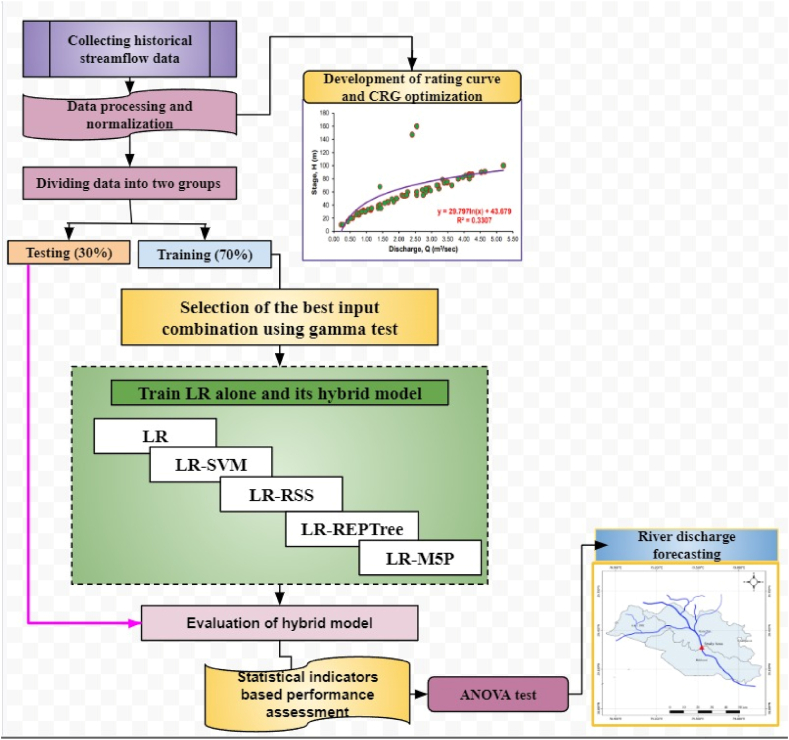
Flowchart of stage-discharge relationship methodology of the study.

In this study, Linear Regression (10.13039/501100009319LR) and its hybrid models, such as Linear Regression-Random Subspace (LR-RSS), Linear Regression-Reduced Error Pruning Tree (LR-REPTree), linear regression-support vector machine (LR-SVM) and linear regression-M5 pruned (LR-M5P) for estimation of stage-discharge relationship were developed. The methodology for analysing the stage-discharge relationship in the selected study case is presented in Fig. 5.

### 3.1 Linear regression (LR)

The linear regression model is one of the most important tools to predicting the value of the dependent output variable, *Y*, based on the independent input variable, *X* [95]. There is a number of statistical and machine learning algorithms that allow the numeric inputs to be converted into numeric outputs, and the best fit straight line to be calculated from the datasets. The accuracy of the linear regression model were measured with most popular least squares method [96]. The generalized equation of linear regression can be written as follows (Eq. (6)) [97]:

(6) $$Y_{i}=\beta_{0}+{\beta_{1}X}_{1}+{\beta_{2}X}_{2}+\ldots +{\beta_{p}X}_{in}+\varepsilon$$

where Y_i_ is the dependent variable, X_i_ is the explanatory variable; $\beta_{0}$ is the Y-intercept (constant) and $\beta_{n}$ is the slope coefficient of each X_i_; ε is the model's error term or residuals.

### 3.2 Support vector machine (SVM)

Support Vector Machine (SVM) creates the decision boundary or best line to separate *n*-dimensional space into different classes. A hyperplane is created when the SVM algorithm finds the points that are at extremes and converts them into hyperplanes. It is a well-known supervised ML algorithm for regression and classification problems [75]. The equation for linear SVM can be written as follows:

(7) $$x_{1},y_{1}\ldots \ldots \ldots .\;.\;.x_{n},y_{n}$$

where $y_{1}$ is either 1 or −1, depending on which class the point $x_{1}$ is linked to. Each $x_{1}$ represents an *n-*dimensional real vector. In Eq. (7), the maximum-margin hyperplane that divides the group of points, $x_{1}$, when $y_{1}=1$ from the group of points when $y_{1}=-1\text{,}$ which is determined to maximize the distance among different points from either group. The hyperplane which satisfies the following equation for a set of points can be written as below:

(8) $$w^{T}x-b=0$$

In Eq. (8) w = normal vector to the hyperplane. The parameter, $\frac{b}{\left\|w\right\|}$ symbolizes the offset of the hyperplane from the source along the normal vector. The parameters selected for implementing the SVM algorithm for stage-discharge modeling are shown in Table 2.

**Table 2:** The parameters of the machine learning algorithm used for stage-discharge modelling.

Model name	Description of parameters
**Random Subspace (RSS)**	Batch size-100, Classifier = REPTree, random seed-1, subspace size = 0. 5, numbers of executions slots = 1, number of iterations = 10
**Reduced Error Pruning Tree (REPTree)**	Batch size-100, Initial count = 0, number of folds = 3, random seed = 1, minimum proportion of the variance = 0.001, minimum number = 2, max depth = 1
**Support Vector Machine (SVM)**	Function estimation- F, Value of the gamma variable −10, Kernel type – RBF_Kernel, Kernel parameter = 0.2
**M5 Pruned (M5P)**	Batch size-100, Minimum number of instances = 4

### 3.3 Reduced error pruning tree (REPTree)

**Fig. 6:**
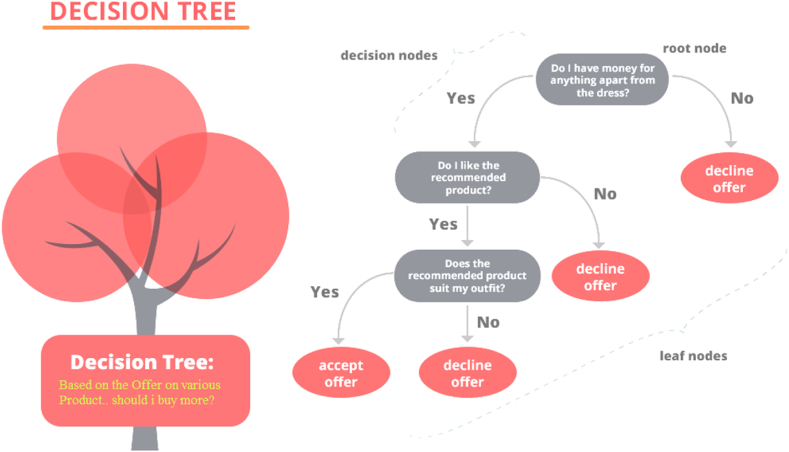
Graphic schematic layout of REPTree.

REPTreeis a data compression type ML technique that decreases the size of decision trees by eliminating unnecessary sections to order the samples. It helps to decrease the complexity of the final classifier, hence raising prognostic precision by reducing overfitting into the dataset, which is the essential benefit of the REPTree method. Backward overfitting is the main responsibility of the pruning operation realized by applying the REPTree model from a computational perspective [77]. This is a fundamental technique of decision tree construction that uses condensed error trimming to construct a regression tree based on variance data, using the REPTree method [98]. The REPTree uses the authenticate dataset to forecast deductive errors accurately [72,99]. Fig. 6 depicts a schematic diagram of the REPTree algorithm and Table 2 indicates the input parameters that were selected for implementing the algorithm for modeling stage-discharge as depicted in Fig. 6.

### 3.4 Random subspace (RSS)

**Fig. 7:**
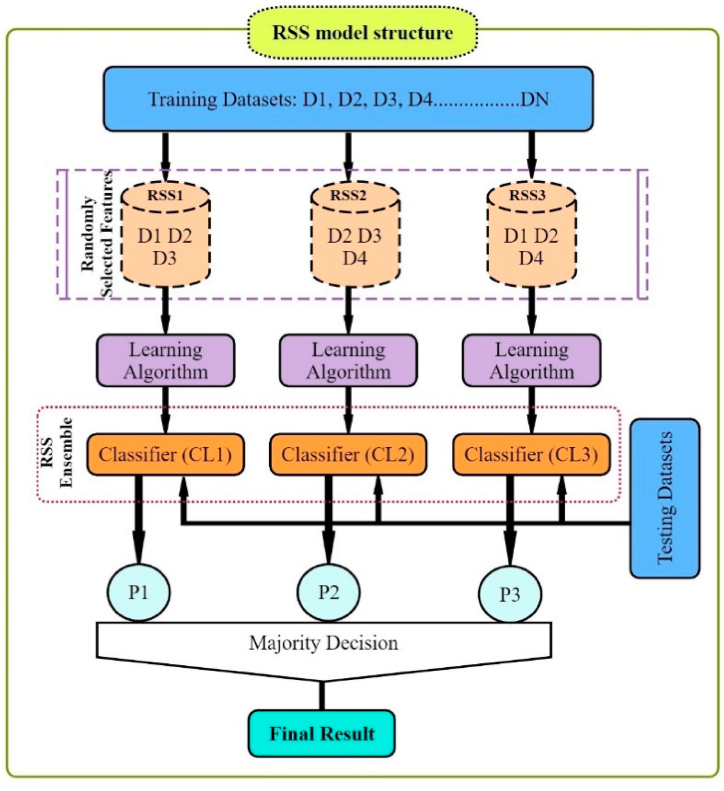
Graphic schematic layout of RSS.

In the context of machine learning, Random Subspace (RSS) is an ML algorithm which combines the prediction variables from a number of decision trees trained on multiple subsets of columns from the training data in order to produce the best prediction results [74]. The problem-independent metaheuristic technique can be applied to a wide range of problems and is a versatile metaheuristic technique [100]. Random Subspace is a particularly effective algorithm when there is a small number of training datasets compared to the amount of data to analyze [75]. This technique introduces randomness into the formulation of issues by selecting certain variables and substituting them at random in a random place [77]. As a robust algorithm, this algorithm combines various weak classifiers in order to produce a robust classifier [101,102]. RSS can be compared to other methods of decision trees, like bagging, in which trees are generated by using samples of the training dataset from a variety of samples of series, such as random forest (RF), which uses ideas from bagging or the random subspace model to generate trees. In spite of decision trees being used in the random subspace model, it can be easily used with any ML model. Depending on the input variables that are used, the performance of the model varies significantly [103]. The first step of the RSS algorithm is to classify the initial space in subsets. Then, the results are attained by the majority of polls using the following Eq. (9):

(9) $$\beta \left(x\right)={\mathrm{argmax}}_{y\in \left\{-1,1\right\}}\sum \delta_{sng}\left(C^{b}\left(x\right)\right),y$$

where δ is the Kronecker delta symbol, y∈{−1,1} is a decision or class label of the classifier, and $C^{b}\left(x\right)$ is the classification integration (C = 1, 2, …). The graphic schematic diagram and parameters selected for implementing the Random Subspace algorithm for stage-discharge modeling are shown in Fig. 7 and Table 2, respectively.

### 3.5 Decision tree with M5P (M5P)

**Fig. 8:**
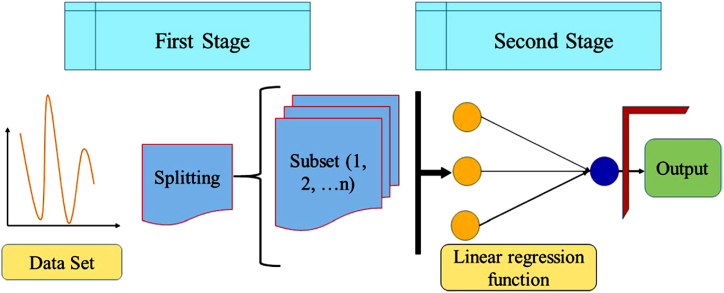
Graphic schematic layout of M5P.

The M5P model tree is a regression model for the continuous variables. It is an improved model of the M5 technique which can efficiently handle large datasets with high dimensionality [77]. M5P follows a multivariate linear regression model to create classification and regression trees through a rapid, simple, and precise procedure. As a result, it reduces the variation of a variable within a specific subspace. The M5P model tree algorithm has two steps: the growing and pruning stages. The nodes are split in the growing stage based on the values of attributes entangled; the main objective is to decrease the prediction error for numerical responses at the terminal nodes and increase the depth of the decision tree. The pruning stage evaluates how much each attribute provides to the prediction error at a node, then cuts off unessential branches. The M5P model has wide applications in hydrology, such as the stage-discharge relationship model [64], streamflow forecasting [104], forecasting for lake level [105], and simulating the rainfall-runoff process [106]. The graphic schematic diagram and parameters selected for implementing the Decision Tree with the M5P algorithm for stage-discharge modeling are shown in Fig. 8 and Table 2, respectively.

### 3.6 Stacked hybridization of the machine learning algorithms

This study used a stack of hybrid algorithms technique to predict the stream discharge of the Gaula Barrage River on a daily basis. Wolpert [107] proposed a technique for stacking hybrid algorithms so-called the stacked hybridization. In the training period, this method provides the favorable environment for ensemble algorithms, i.e., algorithms that can stacked two or more algorithms in a particular sequence. In studies, stacking hybrid algorithms is found to enhance algorithm predictability by improving their efficiency [[108], [109], [110]]. In stacking hybrid generalization, first-level learners are used to process and predict training data sets in order to train and forecast new data set. The first-level learners' projected results were combined to create a new training dataset for the meta-learner, i.e., linear regression-random subspace (LR-RSS), linear regression-reduced error pruning tree (LR-REPTree), linear regression-support vector machine (LR-SVM) and linear regression-M5 pruned (LR-M5P) model. Sikora and Al-Laymoun [111] and Zhou [112] provided more details on stacked hybrid generalization.

## 4 Gamma test (GT)

Hydrological processes are highly complex, dynamic, and non-uniform. A Gamma test establishes an impartial and multi-objective way of determining each input parameter's significant potential. Scholars use a tedious and time-consuming trial-and-error method to determine the ideal input combination. Therefore, to resolve this problem, a novel approach Gamma Test is used to evaluate the ideal input variables in a data set, introduced by Stefansson et al. [113]. It is competent enough to create a trustworthy and smooth model. The two-gamma test statistic, gamma value (Г), and V-ratio are used to select the number of input variables. The relationship between the inputs *(x)* and output *(y)* variables are determined by Eq. (10):

(10) y=Gx+Г

where *G* and Г denote the gradient and intercept of the regression line (*x* = 0), *y* describes the output. Another indicator, i.e., V-ratio (VR) are determined by Eq. (11):

(11) $$VR=\frac{Г}{\sigma^{2}\left(y\right)}$$

here, Г is the gamma function, and *σ*^2^*(y)* is the output variance. In order to find the best possible combination of inputs that produces the minimum absolute Gamma value, one can apply the Gamma test on all possibilities of input combinations. If m scalar inputs exist, 2m-1 potential input combinations. When the *V*-ratio is close to zero, we have a higher chance of model consistency; when the values of gamma, standard error, and *V*-ratio are lower, we can produce a superior mathematical model. The most significant input pairings were chosen according to the lowest values of gamma, standard error, and V-ratio [114].

## 5 Model performance evaluation indices

In order to evaluate the performance and accuracy of the developed models, visual observation was conducted as well as a variety of statistical and hydrological criteria were applied in order to obtain quantitative results. These includes: the Nash Sutcliffe model Efficiency coefficient (NSE), Willmott Index of Agreement (d), Kling-Gupta efficiency (KGE), Mean absolute error (MAE), Mean bias error (MBE), Relative bias in percent (RE), Root Mean Square Error (RMSE) Pearson correlation coefficient (PCC) and coefficient of determination (R^2^). These statistical parameters are summarized in Eq. 12–20. Additional to the statistical parameters stated in Eqs. 14–22, the correctness of the investigated models were validated using Box-and-whisker plots and a Taylor diagram (TD) [115], among other techniques (*i.e.,* time series plot, scatter plot, and relative error). A simplified definition of the Taylor diagram thoroughly depicts the observed and expected data [115]. Taylor delivered a single demonstration demonstrating how to show several assessment metrics in real-time simultaneously. Correlation coefficients and standard deviation values between expected and observed values might be shown in this diagram to aid in the detection of changes between the two values [37,115,116].

**Table:** 

Equation	Range	Ideal value	References	
$MAE=\frac{1}{N}\sum_{i=1}^{N}\left|Q_{i}^{Obs}-Q_{i}^{Cal}\right|$	0 to ∞	0	Tikhamarine et al. [117]	(12)
$MBE=\frac{1}{N}\sum_{i=1}^{N}\left(Q_{i}^{Obs}-Q_{i}^{Cal}\right)$	−∞ to +∞	0	Valipour [118]	(13)
$RE=\frac{\frac{1}{N}\sum_{i=1}^{N}\left(Q_{i}^{Cal}-Q_{i}^{Obs}\right)}{\frac{1}{N}\sum_{i=1}^{N}\left(Q_{i}^{Obs}\right)}$	0 to ∞	0	Walther [119]	(14)
$RMSE=\sqrt{\frac{1}{N}\sum_{i=1}^{N}{\left(Q_{i}^{Obs}-Q_{i}^{Cal}\right)}^{2}}$	0 to ∞	0	Pandey et al. [120]	(15)
$R^{2}=1-\frac{\sum_{i=1}^{N}{\left(Q_{i}^{Obs}-Q_{i}^{Cal}\right)}^{2}}{\sum_{i=1}^{N}{\left(Q_{i}^{Obs}-\bar{Q_{i}^{Cal}}\right)}^{2}}$	0 to 1	1	Nagelkerke [121]	(16)
$PCC=\sqrt{1-\frac{\sum_{i=1}^{N}{\left(Q_{i}^{Obs}-Q_{i}^{Cal}\right)}^{2}}{\sum_{i=1}^{N}{\left(Q_{i}^{Obs}-\bar{Q_{i}^{Cal}}\right)}^{2}}}$	−1 to +1	1	Ozer [122]	(17)
$NSE=1-\frac{\sum_{i=1}^{N}{\left(Q_{i}^{Obs}-Q_{i}^{Cal}\right)}^{2}}{{\left(Q_{i}^{Obs}-\bar{Q_{i}^{Obs}}\right)}^{2}}$	−∞ to 1	1	Nash and Sutcliffe [123,124]	(18)
$d=1-\frac{\sum_{i=1}^{N}{\left(Q_{i}^{Obs}-Q_{i}^{Cal}\right)}^{2}}{\sum_{i=1}^{N}{\left(\left|Q_{i}^{Cal}-\bar{Q_{i}^{Obs}}\right|+\left|Q_{i}^{Obs}-\bar{Q_{i}^{Obs}}\right|\right)}^{2}}$	0 to 1	1	Willmott [125,126]	(19)
$KGE=\sqrt{{\left(PCC\right)}^{2}+{\left(\frac{CD}{RD}\right)}^{2}+{\left(\frac{CM}{RM}-1\right)}^{2}}$	−∞ to 1	1	Gupta et al. [127]	(20)

Note: $Q_{i}^{Cal}$ = ith forecasted discharge data; $Q_{i}^{Obs}$ = observed discharge data; N = number of observations; $\bar{Q_{i}^{Cal}}$ = mean value of observed discharge; $\bar{Q_{i}^{Cal}}$ = mean value of forecasted discharge; CC is the Pearson correlation coefficient value; RM is the average of observed values; CM is the average of forecast values; RD is the standard deviation of observation values; and CD is the standard deviation of forecast values. Following the maximum values of NSE, d, KGE, PCC(r), and R^2^; the minimum values of RMSE, MBE; MAE, and RE were near zero among the all-developed models, the most accurate models were chosen [77,78,128,129].

## 6 Results and discussion

**Fig. 9:**
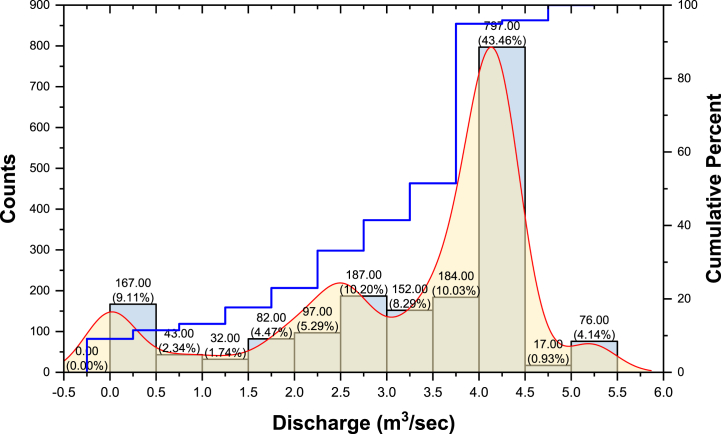
Histogram and kernel distribution of observed discharge at study site.

A preliminary analysis was performed on the dataset by splitting it into 0.5 m^3^/s discharge interval frequency histograms, then observing the data distribution. Accordingly, the data curve under the assumption that the data follow the adaptive kernel density estimation function curve, and the fixed window width kernel density estimation function curve and cumulative percentage are constructed for comparative analysis (Fig. 9). It provides a graphical representation of the distribution of the observed discharge values. This information can be useful in understanding the behavior of the stream or river at the study site and in analyzing and modeling water resources.

It is evident from Fig. 9 that most of the discharge events were 4.0–5.0 m^3^/s (i.e., 43.46% of the total discharge events) during the study period, followed by 2.5–3.0 (i.e., 10.20%) and 3.5–4.0 m^3^/s (i.e.,10.03%). The statistical analysis of daily stage height (m) and discharge (m^3^/s) for the Gaula Barrage is presented in Table 1. Statistical analysis for the datasets collected containing the entire training and testing data sets includes the mean, standard error, median, mode, standard deviation, kurtosis, skewness, minimum, and maximum. From Table 1, one could conclude that the mean of discharge is higher in testing data sets than in training data sets. The standard error of discharge values is higher in training data sets than in testing data sets. The maximum values of the stage in the training data set are higher than that for the testing data set; this may cause difficulty in forecasting discharge at extreme values. However, the maximum and minimum discharge values in the testing set are within the range in the training test, so it may be possible to overcome the problem of estimating extreme discharge values discussed previously.

### 6.1 Conventional method: stage-discharge rating curve

The stage-discharge rating curve parameters *a*, *K*, and *n* were estimated using GRG non-linear optimization method. The results were improved as compared to a simple non-linear model of Eq. (1). Nevertheless, the time needed to obtain optimal parameters by the GRG method was much shorter and easier than the graphical method. The parameters estimated by the non-linear optimization methods as following mathematical relationship were derived:

(21) $$Q=0.03688H^{1.03995}$$

(22) $$Q=0.56293{\left(H-1.02222\right)}^{0.42506}$$

The performance of various parameter estimation methods based on statistical indices was compared to observed and estimated discharges. As a rule of thumb, the estimation method with the lowest error, the highest correlation coefficient, and the model efficiency is considered the best. Table 3 presents the performances of conventional models in estimating discharge at Gaula Barrage.

**Table 3:** Forecasting performance indices of conventional rating curve.

Model	NSE	d	KGE	MAE	MBE	RE	RMSE	PCC	R^2^
Nonlinear rating curve (Eq. (1))	−0.002	0.703	0.514	0.626	−0.055	−5.5%	1.020	0.564	0.318
Rating curve (GRG method) (Eq. (2))	0.389	0.734	0.361	0. 535	0.006	0.6%	0.796	0.731	0.534

**Fig. 10:**
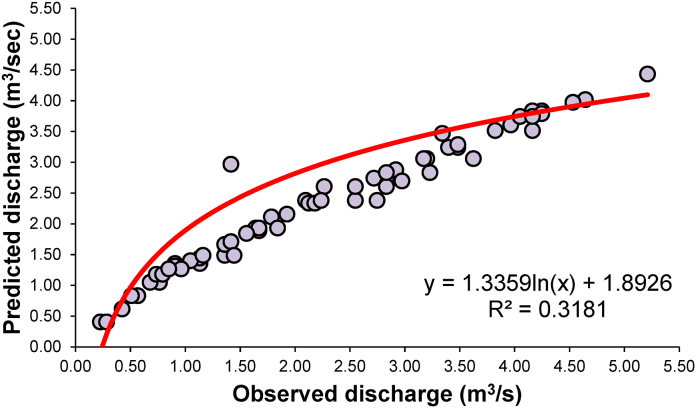
Comparison between predicted and observed discharge and best fit lines for the stage-discharge rating curve using equation (1).

**Fig. 11:**
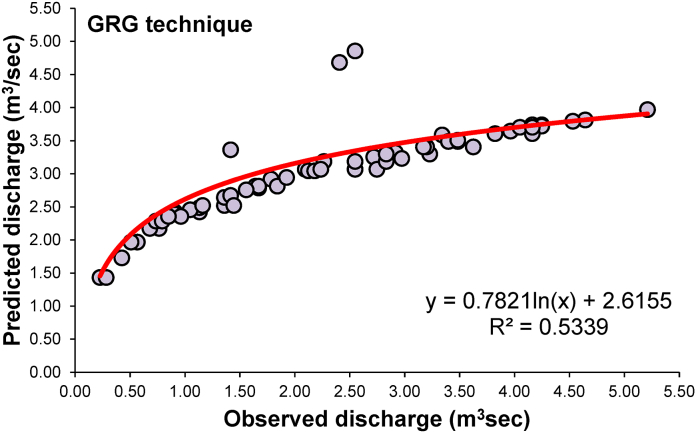
Comparing predicted and observed discharge and best fit lines for the stage-discharge rating curve using the GRG technique (equation (2)).

The conventional model resulted NSE = −0.002, d = 0.703, KGE = 0.514, MAE = 0.626, MBE = −0.055, RE = - 5.5%, RMSE = 1.020, PCC = 0.564 and R^2^ = 0.318 by equation (1). Here, the modified stage-discharge rating curve using GRG optimization algorithms was found to be better than other equation (1) models with NSE = 0.389, d = 0.734, KGE = 0.361, MAE = 0. 535, MBE = 0.006, RE = 0.6%, RMSE = 0.796, PCC = 0.731and R^2^ = 0.534. Fig. 10, Fig. 11 display the observed and simulated discharge values obtained by the conventional stage-discharge rating curve models of equations (21), (22)), respectively, for the study site in scatter plots.

The values of error were observed to be the highest. At the same time, the efficiency was lowest for Equation (1) during estimation, which may be attributed to bias introduced due to logarithmic transformation. Another biggest problem in the non-linear model is that if the data has zero value, it is difficult to convert it to a natural log (logarithm). Some of the data had zero discharge due to the Gaula barrage not being a Perennial River which is a big problem for calculation. In such a case, removing zeros is a big problem and the biggest drawback.

### 6.2 Selection of best input combination for machine learning model

The selection of the optimal input variables is a crucial stage in modeling for the best result of the chosen models. Various input combinations of discharge and stage with multi-lag were used to determine the best input combination for stage and discharge prediction. Various input variables were employed to effectively predict the daily river discharge values in Table 4, following the significant correlation between the inputs and output illustrated above.

**Table 4:** Selection of the best input combination for stage-discharge modeling using gamma test.

Model No.	Model Input Combination	Mask	Gamma value	V-ratio	Standard Error
M1	H_(t)_	1	−0.00355	−0.00420	0.04888
M2	Q_(t-1)_	1000	0.21723	0.86894	0.12072
M3	H_(t-1)_	10	0.09970	0.39878	0.13718
M4	H_(t-2)_	100	0.22233	0.88932	0.12306
M5	Q_(t-2)_	10000	0.19905	0.79618	0.13641
M6	H_(t)_, Q_(t-1)_	10010	−0.00618	−0.02473	0.00964
M7	H_(t)_, Q_(t-2)_	10001	−0.00837	−0.03347	0.00826
M8	H_(t)_, H_(t-1)_	11	−0.00378	−0.01512	0.00624
M9	H_(t)_, H_(t-2)_	101	−0.00687	−0.02748	0.00669
M10	Q_(t-1)_, H_(t-2)_	1100	0.14441	0.57766	0.01595
M11	Q_(t-1)_, H_(t-1)_	1010	0.14324	0.57298	0.12913
M12	Q_(t-2)_, H_(t-1)_	10010	0.11259	0.45034	0.02556
M13	Q_(t-2)_, H_(t-2)_	10100	0.18501	0.74005	0.11421
M14	H_(t-1)_, H_(t-2)_	110	0.14911	0.59646	0.02773
M15	H_(t-2)_, Q_(t-2)_, H_(t-1)_	10110	0.09782	0.39129	0.02599
M16	H_(t-2)_, Q_(t-2)_, Q_(t-1)_	11100	0.10623	0.42491	0.02526
M17	H_(t-1)_, H_(t-2)_, Q_(t-1)_	1110	0.14737	0.58946	0.01398
M18	Q_(t-1)_, Q_(t-2)_, H_(t-1)_	11010	0.11715	0.46861	0.02302
M19**	H_(t)_, Q_(t-2)_, H_(t-2)_	10101	−0.01025	−0.04099	0.01149
M20	H_(t)_, H_(t-1)_, H_(t-2)_	111	−0.00591	−0.02365	0.00757
M21	H_(t)_, H_(t-1)_, Q_(t-2)_	10011	−0.00684	−0.02737	0.00751
M22	H_(t)_, H_(t-1)_, Q_(t-1)_	1011	−0.00872	−0.03488	0.00927
M23	H_(t)_, H_(t-2)_, Q_(t-1)_	1101	−0.00311	−0.01244	0.00433
M24*	H_(t)_, Q_(t-2)_, Q_(t-1)_	11001	−0.01142	−0.04567	0.00898
M25	Q_(t-1)_, Q_(t-2)_	11000	0.11881	0.47524	0.02353
M26	H_(t)_, H_(t-2)_, H_(t-1)_, Q_(t-1)_	1111	−0.00274	−0.01097	0.00581
M27	H_(t)_, H_(t-2)_, H_(t-1)_, Q_(t-2)_	10111	−0.00527	−0.02107	0.00745
M28	H_(t)_, Q_(t-1)_, Q_(t-2)_, H_(t-1)_	11011	−0.00872	−0.03487	0.00783
M29	H_(t)_, Q_(t-1)_, Q_(t-2)_, H_(t-2)_	11101	−0.00401	−0.01604	0.00640
M30	H_(t-1)_, H_(t-2)_, Q_(t-2)_, Q_(t-1)_	11110	0.10364	0.41457	0.02555
M31	H_(t)_, H_(t-1)_, H_(t-2)_, Q_(t-2)_, Q_(t-1)_	11111	−0.00393	−0.01571	0.00628

Note: *Chosen as input combination 1 and **Choose as input combination 2.

The gamma test was used to compare the relative performance of various possible combinations to choose the best input sequence for creating the stage-discharge relationship prediction model. Table 4 lists the values of the three gamma test indicators mask, gamma value, and V ratio, along with the standard error for each of the 31 input pairings. The Mask is displayed using five digits that correspond to the five variables that were taken into consideration in this study to choose inputs: H_(t)_, H_(t-1)_, H_(t-2)_, and Q_(t-2)_ Q_(t-1)_. Digit “1″ denotes an input being utilized, whereas “0″ denotes an input not being used. Hence, “10000″ implies that only H_(t)_ is utilized as an input, while “11111″ denotes that all parameters are used as input. The lower gamma test statistics show that an input combination performs better. Out of 31 feasible combinations, model numbers 24 (input combination-1) and 19 (input combination-2) were picked as the best and second-best input combinations for further study at Gaula Barrage.

### 6.3 Quantitative and qualitative assessment of machine learning models

The best input combination has been selected using the nine statistical parameter indices. For input combination one, *i.e.,* model M24, whose input was H_(t)_, Q_(t-2)_, Q_(t-1)_, Table 5 summarizes the values of nine performance parameters for models using different machine learning techniques in the training and testing dataset. It was observed from Table 5 that the LR-REPTree model was found to be better for forecasting the discharge during both the training and testing periods. The LR-REPTree model had a maximum value of NSE = 0.995, d = 0.999, KGE = 0.996, PCC(r) = 0.998, and R^2^ = 0.996 and minimum value of RMSE = 0.094, MAE = 0.033, MBE = −0.001 and RE = −0.1% during training data set, while in testing data set LR-REPTree model had a maximum value of NSE = 0.993, d = 0.998, KGE = 0.987, PCC(r) = 0.997, and R^2^ = 0.994 and minimum value of RMSE = 0.109, MAE = 0.041, MBE = −0.010 and RE = −0.1%, respectively.

**Table 5:** Forecasting performance indices of models for combination 1.

Model	NSE	d	KGE	MAE	MBE	RE	RMSE	PCC	R^2^
	Training Data set (N = 1284)								
Linear Regression	0.700	0.899	0.743	0.459	−0.009	−0.900%	0.757	0.837	0.701
LR-SVM	0.716	0.907	0.758	0.451	−0.009	−0.900%	0.736	0.847	0.717
LR-RSS	0.936	0.982	0.905	0.177	0.002	0.200%	0.348	0.969	0.939
LR-REPTree	0.995	0.999	0.996	0.033	−0.001	−0.100%	0.094	0.998	0.996
LR-M5P	0.771	0.922	0.740	0.439	−0.016	−1.600%	0.660	0.887	0.787
	**Testing Data set (N = 550)**								
Linear Regression	0.760	0.922	0.773	0.420	−0.104	−10.400%	0.664	0.878	0.771
LR-SVM	0.781	0.930	0.789	0.403	−0.109	−10.900%	0.635	0.890	0.792
LR-RSS	0.934	0.982	0.910	0.179	0.179	0.100%	0.349	0.968	0.937
LR-REPTree	0.993	0.998	0.987	0.041	−0.010	−1.000%	0.109	0.997	0.994
LR-M5P	0.810	0.937	0.753	0.412	−0.132	−13.200%	0.591	0.917	0.841

**Fig. 12:**
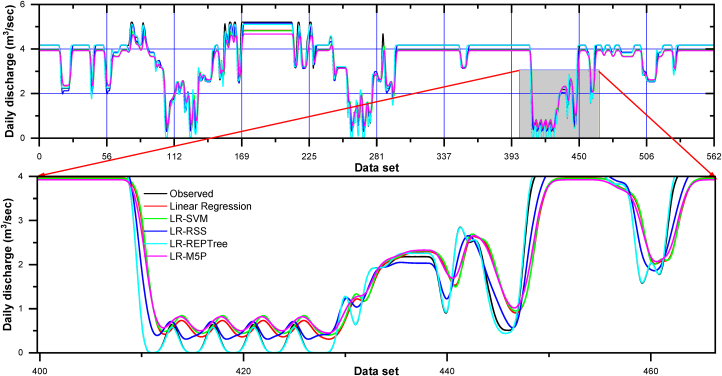
Comparison of daily stage discharge estimation obtained with inputs combination 1 using linear regression, LR-SVM, LR-RSS, LR-REPTree, LR-M5P in testing data sets.

**Fig. 13:**
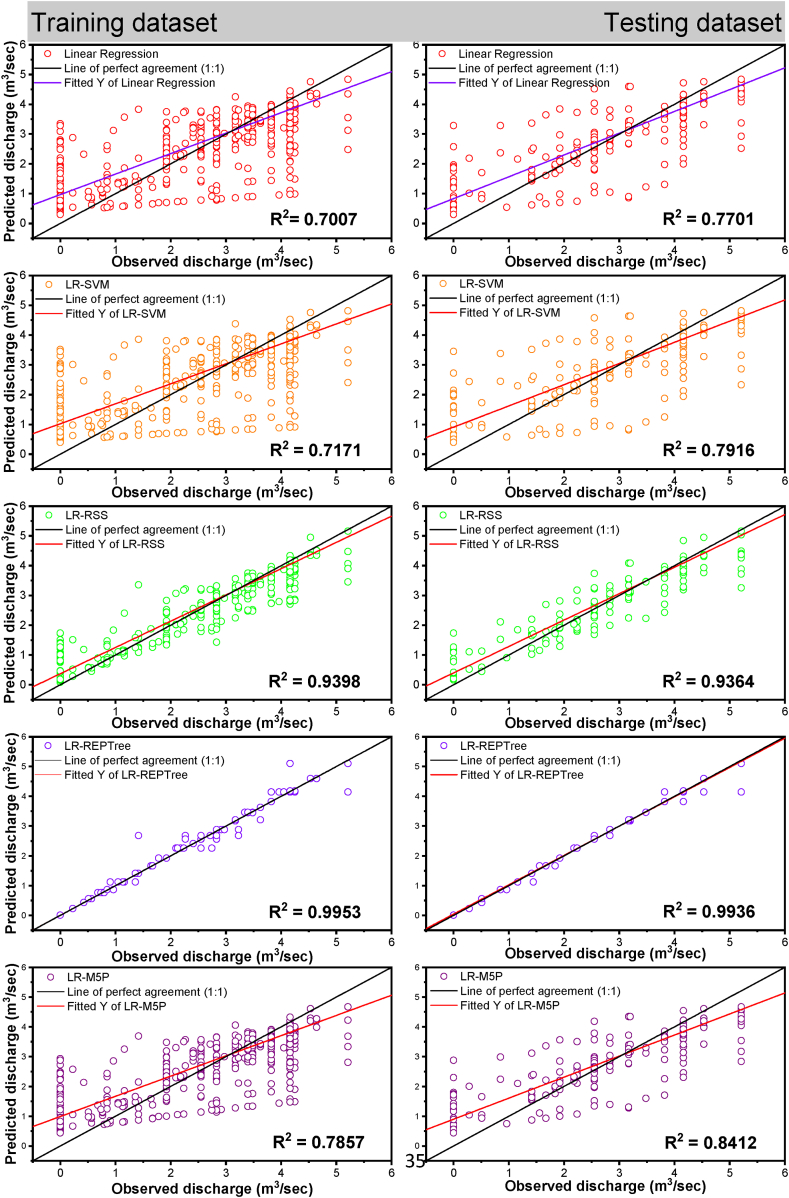
Scatter plots-based comparing all the techniques outcomes vs. observed discharge for input combination 1.

Moreover, LR-RSS ranked the second best for predicting the daily discharge at Gaula Barrage with NSE = 0.936, d = 0.982, KGE = 0.905, PCC(r) = 0.969, and R^2^ = 0.939 and minimum value of RMSE = 0.348, MAE = 0.177, MBE = 0.002 and RE = 0.2%, respectively in training data and NSE = 0.934, d = 0.982, KGE = 0.910, PCC(r) = 0.968, and R^2^ = 0.937 and minimum value of RMSE = 0.349, MAE = 0.179, MBE = 0.179 and RE = 0.1%, respectively in testing data. The visual representation of the observed and the predicted discharge values throughout the training (left side) and testing (right side) dataset in the form of time series (line diagram) and scatter plot for combination 1 of linear regression, LR-SVM, LR-RSS, LR-REPTree, and LR-M5P models are shown in Fig. 12, Fig. 13 respectively.

**Fig. 14:**
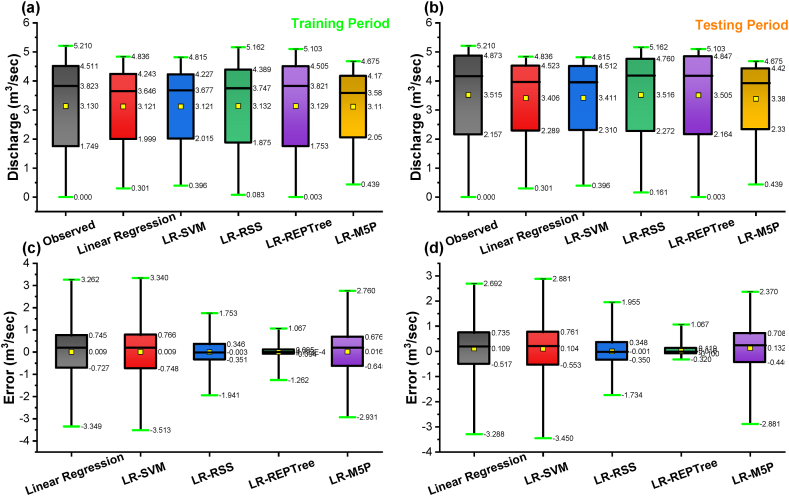
Box-and-whisker plots based on the discharge and the error: (a and c) training and (b and d) testing stages, respectively, for input combination 1.

In nearly all simulations, the expected values were gently under-predicted except in the LR-RSS model. The R^2^ was the highest for the LR-REPTree model (0.996), followed by LR-RSS (0.939), LR-M5P (0.787), LR-SVM (0.717), and linear regression (0.701) in the training period and R^2^ was the highest for the LR-REPTree model (0.994), followed by LR-RSS (0.937), LR-M5P (0.841), LR-SVM (0.792) and linear regression (0.771) in the testing period. Further, for input combination 1, the Box-and-whisker plots are based on the discharge and the error of the various machine learning algorithms, as shown in Fig. 14(a–d), during both the training and testing stages. LR-REPTree is clearly in better agreement with observed data compared to other models. The predictions are more accurate than those of other models followed by LR-RSS, LR-M5P, LR-SVM, and linear regression models.

For input combination two, *i.e.,* model M19, whose input was H_(t)_, Q_(t-2)_, and H_(t-2)_, it can be seen from Table 6 summarizes the values of nine performance parameters for models using different machine learning techniques in the training and testing dataset.

**Table 6:** Forecasting performance indices of models for combination 2.

Model	NSE	d	KGE	MAE	MBE	RE	RMSE	PCC	R^2^
	Training Data set (N = 1284)								
Linear Regression	0.638	0.855	0.607	0.598	−0.014	−1.400%	0.830	0.815	0.664
LR-SVM	0.660	0.874	0.665	0.548	−0.012	−1.200%	0.805	0.819	0.671
LR-RSS	0.849	0.955	0.840	0.290	0.009	0.900%	0.536	0.924	0.854
LR-REPTree	0.993	0.998	0.995	0.042	0.006	0.600%	0.119	0.996	0.992
LR-M5P	0.964	0.990	0.933	0.137	0.002	0.200%	0.261	0.983	0.966
	**Testing Data set (N = 550)**								
Linear Regression	0.705	0.887	0.635	0.563	−0.162	−16.200%	0.737	0.875	0.766
LR-SVM	0.734	0.906	0.700	0.503	−0.136	−13.600%	0.700	0.875	0.766
LR-RSS	0.865	0.961	0.864	0.278	−0.015	−8.400%	0.498	0.932	0.869
LR-REPTree	0.941	0.984	0.923	0.143	−0.089	−0.900%	0.331	0.973	0.947
LR-M5P	0.950	0.986	0.892	0.181	−0.084	−1.500%	0.304	0.980	0.960

**Fig. 15:**
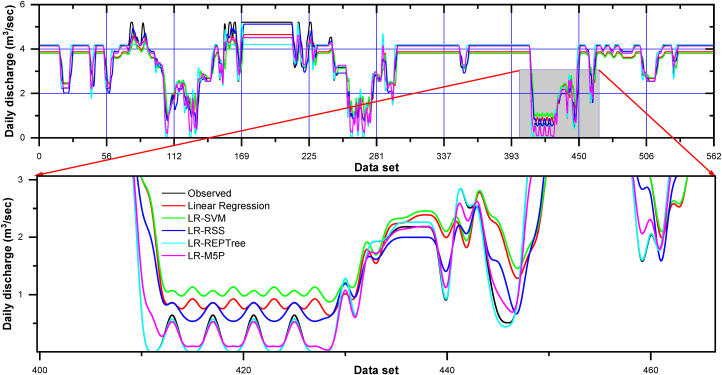
Comparison of daily stage discharge estimation obtained with inputs combination 2 using linear regression, LR-SVM, LR-RSS, LR-REPTree, LR-M5P in testing data sets.

**Fig. 16:**
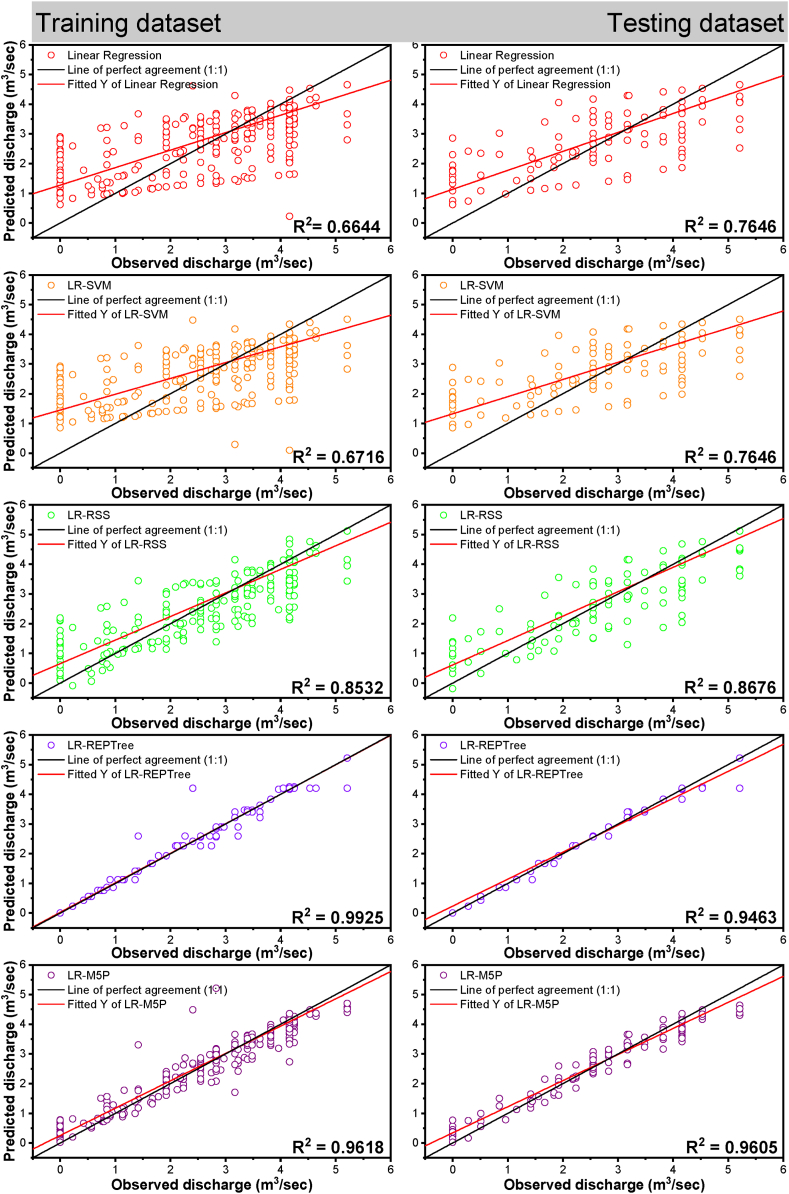
Scatter plots-based comparing all the techniques outcomes vs. observed discharge for input combination 2.

**Fig. 17:**
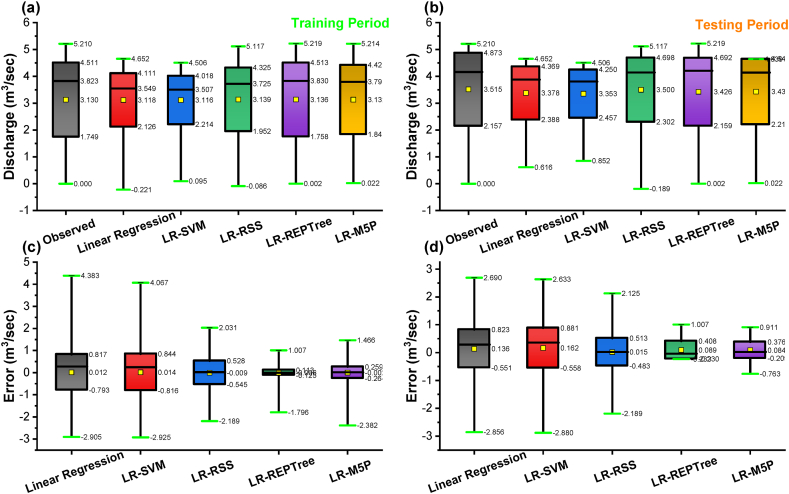
Box-and-whisker plots based on the discharge and the error: (a and c) training and (b and d) testing stages, respectively for input combination 2.

It was observed from Table 6 that the LR-REPTree model was found to be better for forecasting the discharge during both the training and testing periods. The LR-REPTree model had maximum value of NSE = 0.993, d = 0.998, KGE = 0.995, PCC(r) = 0.996, and R^2^ = 0.992 and minimum value of RMSE = 0.119, MAE = 0.042, MBE = −0. 006 and RE = −0.6% during the training data set, while in the testing data set LR-REPTree model had a maximum value of NSE = 0.941, d = 0.984, KGE = 0. 923, PCC(r) = 0. 973, and R^2^ = 0. 947 and minimum value of RMSE = 0. 331, MAE = 0.143, MBE = −0.089 and RE = −0.9%, respectively. Moreover, LR-M5P ranked the second best for predicting the daily discharge at Gaula Barrage with NSE = 0.964, d = 0.990, KGE = 0.933, PCC(r) = 0.983, and R^2^ = 0.966 and minimum value of RMSE = 0.261, MAE = 0.137, MBE = 0.002 and RE = 0.2%, respectively in training data and NSE = 0.950, d = 0.986, KGE = 0.892, PCC(r) = 0.980, and R^2^ = 0.960 and minimum value of RMSE = 0.304, MAE = 0.181, MBE = −0.084 and RE = −1.5%, respectively in testing data. The visual representation of the observed and the predicted discharge values throughout the training and testing dataset in the form of time series (line diagram) and scatter plot for combination 2 of linear regression, LR-SVM, LR-RSS, LR-REPTree, and LR-M5P models are shown in Fig. 15, Fig. 16 respectively. In nearly all simulations, the expected values were gently under-predicted. The R^2^ was the highest for the LR-REPTree model (0.992), followed by LR-M5P (0.966), LR-RSS (0.854), LR-SVM (0.671), and linear regression (0.664) in the training period and R^2^ was the highest for the LR-REPTree model (0.947), followed by LR-M5P (0.960), LR-RSS (0.969), LR-SVM (0.766) and Linear Regression (0.766) in the testing period. Further, for input combination 2, the Box-and-whisker plots are based on the discharge and the error of the various machine learning algorithms, as shown in Fig. 17(a–d), during both the training and testing stages. LR-REPTree is clearly in better agreement with observed data compared to other models. The predictions are more accurate than those of other models followed by LR-M5P, LR-RSS, LR-SVM, and linear regression models.

**Fig. 18:**
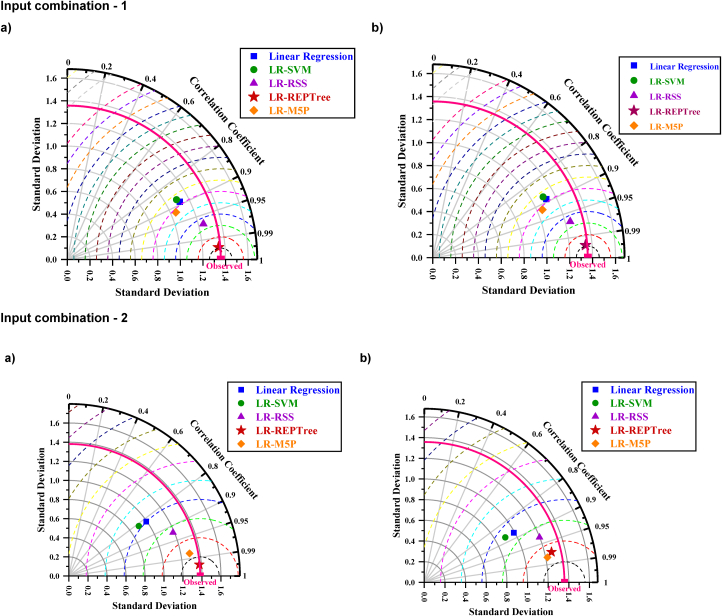
Taylor diagrams of the observed data set and the estimations of the applied soft computing models: (a) training and (b) testing stages for input combination 1 and 2.

Residuals of the LR-REPTree model in both best combinations 1 and 2 were the smallest among all other models (Fig. 13, Fig. 16, respectively). The discharge value was very close to the line of perfect agreement (line 1:1) in the scatter plot. Further, the models' performances were evaluated using the Taylor diagram, as shown in Fig. 18(a and b), throughout the training and testing periods for input combinations 1 and 2, respectively. Based on the standard deviation and correlation, it is evident from Fig. 18 that the LR-REPTree model was closest to the observed location, followed by the LR-RSS model. In the study area, the estimation of the daily discharge was performed using the LR-SVM model, which was the furthest away and produced the worst results. The sequence of models results from best to poor in order LR-REPTree > LR-RSS > LR-M5P > LR-SVM > linear regression for the input combination one and LR-REPTree > LR-M5P > LR-RSS > LR-SVM > linear regression for the input combination 2 for Gaula Barrage site. Hence, the LR-REPTree model can estimate stage-discharge for the Gaula Barrage site.

This study aimed to compare the effectiveness of several machine learning techniques for predicting daily river discharge. The forecasting techniques studied comprise the LR, LR-SVM, LR-RSS, LR-REPTree, and LR-M5P methods. In order to minimize and lessen the effects of flooding on the river's downstream area, this model could be used in real-time short-term flood forecasting models and early warning systems. Additionally, without additional hydrological and meteorological parameters, the suggested models can accurately anticipate the river level using only the previously recorded water level and discharge as inputs. Al-Abadi [130] reported that the stage-discharge relationship could be accurately predicted using backpropagation artificial neural networks, M5 decision trees, and Takagi–Sugeno inference system methods. It was found that the high values of the R^2^ were 0.82, 0.88, and 0.88, respectively, which were significantly less than the obtained values in our present study. Birbal et al. [65] used the gene expression programming method to calculate the river stage-discharge relationship in another study. They found that the maximum R^2^ value was 0.99, which agrees with the findings of the current investigation.

Hence, it can be concluded that machine-learning algorithms can accurately predict future river water-level changes in a shorter time and with fewer inputs. Overall, it can be said that the LR-REPTree model has outperformed other selected and conventional model structures in terms of performance criteria.

### 6.4 Comparison of models

Comparing the machine learning-based models with the old conventional models shows that these models outperformed the conventional rating curve (Table 3, Table 5, and Table 6). A comparison of machine learning-based models indicates that LR-REPTree models work better than other machine learning-based models and conventional rating curves. Table 7 is summarized the result of the ANOVA summary with the sum, average, and variance. Based single-factor ANOVA results (Table 8) suggest that F-value (0.695327541) was less than f-critical (2.960415 (α = 0.001) & 1.831172 (α = 0.05)) and P-value (0.729832) was greater than 0.05 suggesting that difference in predicted values of LR, LR-RSS, LR-SVM, LR-M5P, and LR-REPTree model and actual value were insignificant. This study also compared the effect of input combination; for that, we selected two types of input combination, *i.e.,* best one- and second best. Single-factor ANOVA results (Table 9) for combinations 1 and 2 show that F-values were less than f-critical. P-values were greater than 0.05, suggesting that the difference in the estimated values of LR, LR-RSS, LR-SVM, LR-M5P, and LR-REPTree values is also insignificant.

**Table 7:** Result of ANOVA summary.

Groups	Sum	Average	Variance
Observed	5956.49551	3.24959	1.91354
LR (Combination 1)	5883.80419	3.20993	1.26772
LR (Combination 2)	5864.65119	3.19948	0.99513
LR-RSS (Combination 1)	5959.91471	3.25145	1.59681
LR-RSS (Combination 2)	5958.63847	3.25076	1.43973
LR-SVM (Combination 1)	5886.28726	3.21129	1.23293
LR-SVM (Combination 2)	5847.06714	3.18989	0.81999
LR-M5P (Combination 1)	5862.24404	3.19817	1.12195
LR-M5P (Combination 2)	5912.51879	3.22560	1.62385
LR-REPTree (Combination 1)	5950.53851	3.24634	1.88941
LR-REPTree (Combination 2)	5914.18997	3.22651	1.82004

**Table 8:** Result of ANOVA Single Factor Test for all models.

Groups	F	P-value	F-critical	The difference in predicted values
All models (Between Groups)	0.695327541	0.729832	2.960415 (α = 0.001)	Insignificant
			1.831172 (α = 0.05)

**Table 9:** Comparison results of Single-Factor ANOVA test for LR, LR-RSS, LR-SVM, LR-M5P and LR-REPTree approaches between input combination 1 and 2.

Groups	F	P-value	F-critical	The difference in predicted values
LR	0.085246572	0.770325964	3.843996651	Insignificant
LR-RSS	0.000212683	0.988365124	3.843996651	Insignificant
LR-SVM	0.397890221	0.52822	3.843996651	Insignificant
LR-M5P	0.49214918	0.483014698	3.843996651	Insignificant
LR-REPTree	0.193728918	0.659857	3.843996651	Insignificant

## 7 Conclusions

As flooding causes a high level of human and financial loss, it is necessary to obtain the design discharge of the rivers in order to design these structures. Predicting daily, weekly, and monthly discharges during extreme events such as floods and droughts is vital. Thus, the current investigation was designed to forecast the daily discharge at Gaula Barrage, Uttarakhand, by employing GRG, LR, LR-RSS, LR-REPTree, LR-SVM, and LR-M5P models. Stage discharge-rating curves were developed for the study sites using non-linear regression and non-linear optimization methods such as GRG solver. Values of statistical indices clearly show the superiority of GRG non-linear optimization methods over the conventional rating curve method. Despite this, among all non-linear optimization methods, the GRG technique has proven powerful, easy, and promising for predicting parameter values of non-linear equations such as stage-discharge relationships. The gamma test analysis gives an appropriate idea to select the best combination of input parameters in time series-based modeling.

Based on statistical performance indicators and visual examination, the results exposed that the LR-REPTree model with H_(t)_, Q_(t-2)_, Q_(t-1)_ for combination one and H_(t)_, Q_(t-2)_, H_(t-2)_ for combination two inputs perform superior to the GRG, LR, LR-RSS, LR-SVM and LR-M5P models for daily discharge forecasting during monsoon season at the study site. For future modeling using highly variable discharge data, researchers can benefit from the best performance of the LR-REPTree technique. The ANOVA Single Factor Test also confirms that the predictions for all models of machine landings are very close and not significantly different. The models' performance was very good, even in the best and second-best input selection, and close to the observed value. i.e., insignificant. Zero values in observed data are a serious problem in non-linear models in the stage-discharge rating curve. Thus, machine learning is a good way to tackle this problem. Moreover, it is recommended that researchers avoid biases associated with overestimations and underestimations when dealing with highly variable data to the best of their abilities.

## Author contribution

Dinesh Kumar Vishwakarma: Conceived and designed the experiments; Performed the experiments; Analyzed and interpreted the data; Contributed reagents, materials, analysis tools or data; Wrote the paper.

Alban Kuriqi, Nadhir Al-Ansari & Salwan Ali Abed: Analyzed and interpreted the data; Wrote the paper.

Gottam Kishore, Nadhir Al-Ansari & Pravendra Kumar: Conceived and designed the experiments; Contributed reagents, materials, analysis tools or data; Wrote the paper.

Kusum Pandey, Nand Lal Kushwaha & Arif Jewel: Performed the experiments; Analyzed and interpreted the data; Contributed reagents, materials, analysis tools or data; Wrote the paper.

## Funding

No funding was received for conducting this study.

## Data availability statement

Data will be made available on request.

## Ethical approval

All authors comply with the journal Stochastic Environmental Research and Risk Assessment guidelines.

## Consent to participate

All authors agreed to participate in this study.

## Consent to publication

All authors agreed to the publication of this manuscript.

## Declaration of competing interest

The authors declare that they have no known competing financial interests or personal relationships that could have appeared to influence the work reported in this paper.
